# Movement Demands of Elite U20 International Rugby Union Players

**DOI:** 10.1371/journal.pone.0153275

**Published:** 2016-04-07

**Authors:** Daniel Cunningham, David A. Shearer, Scott Drawer, Robin Eager, Neil Taylor, Christian Cook, Liam P. Kilduff

**Affiliations:** 1 Applied Sport Technology Exercise and Medicine Research Centre (A-STEM), College of Engineering, Swansea University, Swansea, Wales; 2 Department of Psychology, University of South Wales, Rhondda Cynon Taff, Wales; 3 The Rugby Football Union, Greater London, England; Norwegian University of Science and Technology, NORWAY

## Abstract

The purpose of this study was to quantify movement demands of elite international age grade (U20) rugby union players during competitive tournament match play. Forty elite professional players from an U20 international performance squad were monitored using 10Hz global positioning systems (GPS) during 15 international tournament matches during the 2014/15 and 2015/16 seasons. Data on distances, velocities, accelerations, decelerations, high metabolic load (HML) distance and efforts, and number of sprints were derived. Data files from players who played over 60 min (n = 161) were separated firstly into Forwards and Backs, and more specifically into six positional groups; FR—Front Row (prop & hooker), SR—Second Row, BR—Back Row (Flankers & No.8), HB—Half Backs (scrum half & outside half), MF—Midfield (centres), B3 –Back Three (wings & full back) for match analysis. Analysis revealed significant differences between forwards and backs positions. Backs scored higher on all variables measured with the exception of number of moderate accelerations, decelerations (no difference). The centres covered the greatest total distance with the front row covering the least (6.51 ± 0.71 vs 4.97 ± 0.75 km, p < 0.001). The front row also covered the least high speed running (HSR) distance compared to the back three (211.6 ± 112.7 vs 728.4 ± 150.2 m, p < 0.001) who covered the most HSR distance, affirming that backs cover greater distances but forwards have greater contact loads. These findings highlight for the first time differences in the movement characteristics of elite age grade rugby union players specific to positional roles.

## Introduction

Rugby Union is an intermittent high intensity invasion game, involving periods of static exertions, collisions and running, interspersed with variable periods of lower intensity work and rest [1–3]. Micro sensor technology (e.g. GPS) is currently used widely in team sports to quantify the workloads of players during training and matches at the elite level of the game [1, 2, 4–6]; this technology enhances the ability to characterise a player’s movement patterns in rugby union, allowing coaches a greater insight into the positional requirements of performance [2, 6]. This in turn may facilitate the planning, implementation and monitoring of training programmes in order to achieve the desired physiological stress while minimising the risk of overtraining and injury [7, 8].

A number of recent studies have reported the movement patterns of senior professional rugby union players (e.g. 1, 2, 6) and also evaluated the physiological stress [4, 6] and recovery time-frames as a result of match-play [4]. Jones and co-workers [4] have shown that forwards and backs covered 60.4 ± 7.8 m•min^-1^ and 67.8 ± 8.2 m•min^-1^ respectively during 4 European Cup games. These researchers also reported moderate and moderate-large effect size correlations for forwards and backs between number of impacts (as identified by performance analysis) and changes in creatine kinase (CK) concentrations. Moderate effect size correlations were also reported for high speed running (HSR) distance and changes in CK concentrations for backs only, identifying the potential need for tailored recovery and training strategies.

However, compared with senior rugby, there is far less information describing match demands of age-grade Internationals. Greater knowledge of the demands of age-grade international games will allow coaches to evaluate movement characteristics and identify where it fits in the development plan for the players. It would be useful, for example, for coaches to know if the demands are high enough to prepare players for senior international rugby or premiership level at their respective clubs, and if not for them to provide appropriate exposure to certain demands during their long term plans to support development. Quantification of the movement demands and activities performed during match play is commonly referred to as time motion analysis (TMA) [9]. Hartwig et al., [10] monitored 118 adolescent male rugby players (14–18 years old) using two forms of TMA (GPS technology and video based analysis) taken between 2003–08, which included 116 training sessions and 53 games. They reported that forwards and backs covered distances of 3,795 ± 565 m and 4,140 ± 460 m per hour of match play, equating to ~ 63 m•min^-1^ and 69 m•min^-1^ for forwards and backs respectively. These values are similar to those reported in senior professional northern hemisphere rugby (60.4 and 67.8 m•min^-1^ [4] and 60.9 and 67.9 m•min^-1^ [1]), but not to values recently reported from a senior professional southern hemisphere team (77.3 and 84.7 m•min^-1^) for forwards and backs respectively [6]. It is important to note that the data of Hartwig and colleagues [10] is 7–12 years old and differences may just reflect the changing demands of the game over time. In addition, caution must be used when comparing these results as Randers et al., [11] reported large between-system differences in the determination of the distances covered between video based TMA and GPS. Given that there is no literature on GPS use at U20 level, it is currently not possible to compare movement demands. Additionally within the forwards and backs playing groups there are distinct playing positions that each have both unique and overlapping demands [1]. Therefore, more detailed and applicable information on playing demands would be deduced from analysing in more detail the playing groups (i.e. Front Row, Second Row, Back Row, Half Backs, Midfield/Centres & Back Three).

Given the differences between physical characteristics of senior and junior players, it is pertinent to investigate whether these differences manifest themselves in the movement demands (locomotive aspects) of the game. Due to the lack of available GPS data in age-grade rugby, no comparisons to seniors has been possible until now. Current LTAD models identify that younger athletes need to be trained differently to their senior counterparts [12] due to the different levels of anthropometric, strength and power capabilities [13–15], hence it would be unwise to use senior data as a model for current U20 players. Therefore, the aim of this study was to characterise the locomotive match demands of elite U20 international rugby using 10Hz GPS technology.

## Methods

Forty elite professional junior players from an U20 international performance squad participated in the study. Prior to providing written informed consent, participants were given information outlining the rationale, potential applications and procedures associated with the study. Ethical approval was granted by the Swansea University Ethics Committee. All players were considered healthy and injury-free at the time of the study and were in full-time training. The participants (age 19.65 ± 0.47 years, body mass 99.69 ± 11.32 kg, height 185.71 ± 6.85 cm) provided a total of 161 GPS files from 15 games from two 6 Nations tournaments (2014 and 2015) and the 2015 Junior World Cup. Previous studies have shown that substitute players display greater work-rates compared to players who start the match, suggesting that these players do not pace their involvement [16]. Therefore, to be included in the analysis players had to complete ≥60 mins match time [4, 17]. Each player provided at least 1 GPS file with the largest number of files provided by any player being 11.

### Procedures

All Matches took place between January 2014 and June 2015, each player wore a GPS unit (Viper Pod, STATSport, Belfast, UK) in a bespoke pocket incorporated into their playing jersey on the upper thoracic spine between the scapulae to reduce movement artefacts [18]. The GPS units captured data at a sampling frequency of 10Hz. Recent advancements in GPS technology have made 10 Hz units commercially available, which are more accurate for quantifying movement patterns in team sports [19, 20]. For example, Varley et al., [20] reported that a 10 Hz GPS unit was two to three times more accurate for instantaneous velocity during tasks completed at a range of velocities compared to a criterion measure, six times more reliable for measuring maximum instantaneous velocity and had a coefficient of variation less than or similar to the calculated smallest worthwhile change [21] during all phases of acceleration/deceleration. In our study, all participants were already familiarized with the devices as part of their day-to-day training and playing practices. Units were activated according to the manufacturer’s guidelines immediately prior to the pre-match warm-up (~30 minutes before kick-off), and to avoid inter-unit variation players wore the same GPS device for each match. After each match, the raw data files were analyzed and 15 indices of physical performance were derived automatically (Viper PSA software, STATSports, Belfast, UK).

### Locomotor Variables

The total distance (km), distance relative to playing time (m•min^-1^), high speed running (HSR) >18.1km•h^-1^ (the threshold used in numerous rugby GPS studies; e.g. Austin & Kelly [22]), HSR relative to playing time (HSR•min^-1^), number of sprints, number of sprints relative to playing time (sprints•min^-1^), moderate, high and severe intensity accelerations and decelerations (±2-3m•s^-2^, ±3-4m•s^-2^, ±>4m•s^-2^), high metabolic load distance (HML; defined as distance covered accelerating and decelerating over 2 m•s^-2^ and/or distance covered >5 m•s^-1^), and high metabolic load efforts (the number of separate movements/efforts undertaken in producing HML distance). Total time was calculated for ‘playing time’ only, that is, the time the player was on the playing field only, with time off the field (e.g., half time, periods on the bench/sin bin) removed from the data analysis. Time off during match play, such as injury time or video referee, was included in the study, because this was part of the game duration; hence ‘playing time’ may exceed the standard 80 minutes of match play.

### Data analysis

Two separate MANOVA (Pillai’s trace = *V*) for all 15 dependent variables were conducted for each independent variable (i.e., playing position—Forward, Back; position group—FR—Front Row, SR—Second Row, BR—Back Row, HB—Half Backs, MF—Midfield, B3—Back Three). The direction of significant difference for playing position were identified using a combination of univariate comparison and examinations of means. Hochberg post hoc test were used to separate the scores for each position group into homogenous subsets. These subsets indicated both the clear difference between certain position groups and variables in which cross-over between subsets occurred.

## Results

Mean and standard deviations for all variables indicated that all dependent variables values were greater for backs (Table 1). A one-way MANOVA (Pillai’s trace = *V*) revealed an overall significant difference between position types (forwards vs backs) across the 16 dependent variables included in the model (*F* (15, 143) = 34.53, *p* < .001, η^2^ = .79). Test of between subject effects between forwards and backs for each dependent variable revealed significant differences for all but accelerations and decelerations 2-3m•s^-2^ indicating that both groups were similar for these variables.

**Table 1:** Means and Standard Deviations of Playing Groups.

Position Group	Forwards (21) (n = 81)	Backs (19) (n = 80)	FR (8) (n = 26)	SR (5) (n = 24)	BR (8) (n = 31)	HB (5) (n = 15)	MF (7) (n = 29)	B3 (7) (n = 36)
	M ± SD	M ± SD	M ± SD	M ± SD	M ± SD	M ± SD	M ± SD	M ± SD
Total Distance (km)	5.37 ± 0.83*	6.23 ± 0.80	4.97 ± 0.75^c^^d^^e^^f^	5.41 ± 0.48^e^^f^	5.67 ± 0.98^a^^e^	5.84 ± 0.89^a^	6.51 ± 0.71^a^^b^^c^	6.18 ± 0.77^a^^b^
M•min^-1^	61.5 ± 8.0*	69.1 ± 7.6	60.1 ± 7.2^e^^f^	60.8 ± 5.9^e^^f^	63.2 ± 9.8^e^	67.5 ± 9.1	70.5 ± 6.8^a^^b^^c^	68.7 ± 7.6^a^^b^
HSR (m)	284.2 ± 134.9*	656.9 ± 182.7	211.6 ± 112.7^c^^d^^e^^f^	265.3 ± 94.2^d^^e^^f^	359.7 ± 142.7^a^^e^^f^	476.1 ± 204.1^a^^b^^e^^f^	661.7 ± 145.1^a^^b^^c^^d^	728.4 ± 150.2^a^^b^^c^^d^
HSR m•min^-1^	3.2 ± 1.5*	7.3 ± 2.1	2.5 ± 1.3^c^^d^^e^^f^	3.0 ± 1.1^d^^e^^f^	4.0 ± 1.6^a^^e^^f^	5.5 ± 2.4^a^^b^^e^^f^	7.2 ± 1.7^a^^b^^c^^d^	8.1 ± 1.7^a^^b^^c^^d^
Duration (min)	87.6 ± 9.7*	90.4 ± 8.1	83.2 ± 10.9^e^^f^	89.3 ± 7.5	90.0 ± 9.1	87.0 ± 10.6	92.5 ± 6.4^a^	90.1 ± 7.9^a^
HML Distance (m)	701.3 ± 198.7*	1060.4 ± 218.1	584.9 ± 199.1^c^^d^^e^^f^	673.3 ± 124.1^d^^e^^f^	820.6 ± 182.5^a^^e^^f^	954.8 ± 304.0^a^^b^	1103.4 ± 168.6^a^^b^^c^	1069.7 ± 203.0^a^^b^^c^
HML Efforts	78.8 ± 21.5*	98.8 ± 21.7	66.0 ± 22.6^c^^d^^e^^f^	77.2 ± 14.5^d^^e^^f^	90.7 ± 19.0^a^	99.9 ± 27.2^a^^b^	105.0 ± 15.9^a^^b^	93.4 ± 22.4^a^^b^
Accelerations 2-3m•s^-^²	23.6 ± 8.9	26.1 ± 10.1	17.8 ± 6.6^c^^e^^f^	22.9 ± 7.4	29.0 ± 8.5^a^	23.5 ± 13.6	27.4 ± 10.3^a^	26.1 ± 8.1^a^
Accelerations 3-4m•s^-^²	4.3 ± 2.7*	6.4 ± 4.5	3.5 ± 2.0^f^	3.8 ± 2.1^f^	5.5 ± 3.1	4.3 ± 5.4^f^	5.9 ± 2.8	7.6 ± 4.9^a^^b^^d^
Accelerations >4m•s^-^²	0.47 ± 0.84*	0.89 ± 1.37	0.39 ± 0.75^f^	0.25 ± 0.53^f^	0.71 ± 1.04	0.33 ± 0.49^f^	0.45 ± 0.78^f^	1.47 ± 1.73^a^^b^^d^^e^
Decelerations 2-3m•s^-^²	25.2 ± 9.3	25.3 ± 9.3	21.1 ± 9.0^c^	24.8 ± 9.8	28.8 ± 7.9^a^	24.7 ± 9.9	28.1 ± 8.9	23.3 ± 9.1
Decelerations 3-4m•s^-^²	7.5 ± 3.5*	9.5 ± 4.4	6.2 ± 3.7^e^^f^	8.0 ± 3.0^e^	8.2 ± 3.4^e^	6.5 ± 3.6^e^	11.5 ± 4.1^a^^b^^c^^d^	9.2 ± 4.3^a^
Decelerations >4m•s^-^²	2.28 ± 1.65*	4.95 ± 3.0	2.19 ± 1.86^e^^f^	1.58 ± 1.25^e^^f^	2.9 ± 1.54^e^^f^	3.0 ± 1.96^e^^f^	5.24 ± 3.51^a^^b^^c^^d^	5.53 ± 2.62^a^^b^^c^^d^
Sprints	11.15 ± 5.06*	26.44 ± 7.47	8.73 ± 4.52^c^^d^^e^^f^	10.0 ± 3.27^d^^e^^f^	14.07 ± 5.29^a^^e^^f^	17.8 ± 6.47^a^^b^^e^^f^	27.86 ± 6.32^a^^b^^c^^d^	28.89 ± 6.11^a^^b^^c^^d^
Sprint•min^-1^	0.11 ± 0.05*	0.26 ± 0.07	0.09 ± 0.04^c^^d^^e^^f^	0.10 ± 0.03^d^^e^^f^	0.14 ± 0.05^a^^e^^f^	0.18 ± 0.06^a^^b^^e^^f^	0.27 ± 0.06^a^^b^^c^^d^	0.29 ± 0.06^a^^b^^c^^d^

FR = Front Row (Prop & Hooker), SR = Second Row, BR = Back Row, HB = Half Backs, MF = Midfield/Centres, B3 = Back Three (Wing & Fullback). () = Number of players providing GPS files, n = number of GPS files.

* = Significant difference compared to backs group (p <0.05).

^a^ Significant difference compared to FR;

^b^ Significant difference compared to SR;

^c^ Significant difference compared to BR;

^d^ Significant difference compared to HB;

^e^ Significant difference compared to MF;

^f^ Significant difference compared to B3.

A second one-way MANOVA (Pillai’s trace) indicated a significant overall difference for position group across all 15 dependent variables (F (80, 715) = 4.90, p < .001, η^2^ = .35), with between subjects effects indicating that all dependent variables were significantly different across positional groups. Hochberg post-hoc test were used to examine where between group differences (*p* < .05) occurred and to organise data into homogenous subsets for each dependent variable, indicating which position groups had similar (i.e., no significant difference) mean values for each dependent variable. Each dependent variable differed in terms of the number of subsets which explained the data. Cross over existed for all subsets, however where only two subsets occurred cross-over was greatest and in most cases it was difficult to discern any real pattern in the data other than front row forwards never appeared in both subsets indicating they were more distinct than other playing groups. In the one instance when 3 subsets best explained the data, this represented three groups i) front five, ii) back row and half backs, and iii) three quarters (see Table 2). Finally, where four subsets occurred this most often represented i) front five, ii) second row and back row, iii) back row and half-backs, and iv) back-three and midfield.

**Table 2:** Homogenous subsets for all dependent variables measure for U20 sample (*p* < .05).

Dependent variable	Subset 1	Subset 2	Subset 3	Subset 4
Total Distance (km)	FR, SR	SR, BR, HB	BR, HB, B3	B3, MF
M•min^-1^	FR, SR, BR	BR, HB, B3	HB, B3, MF	
HSR (m)	FR, SR	SR, BR	BR, HB	MF, B3
HSR m•min^-1^	FR, SR	SR, BR	HB	MF, B3,
Duration (min)	FR, HB, SR, BR, B3	HB, SR, BR, B3, MF		
HML Distance (m)	FR, SR	SR, BR	BR, HB,	HB, B3, MF
HML Efforts	FR, SR	SR, BR, B3	BR, B3, HB, MF	
Accelerations 2-3m•s^-^²	FR, SR, HB	SR, HB, B3, MF, BR		
Accelerations 3-4m•s^-^²	FR, SR, HB, BR, MF	BR, MF, B3		
Accelerations >4m•s^-^²	SR, HB, FR, MF, BR	BR, B3		
Decelerations 2-3m•s^-^²	FR, B3, HB, SR, MF	B3, HB, SR, MF, BR		
Decelerations 3-4m•s^-^²	FR, HB, SR, BR, B3	B3, MF		
Decelerations >4m•s^-^²	SR, FR, BR, HB	MF, B3		
Sprints	FR, SR	SR, BR,	BR, HB	MF, B3
Sprint•min^-1^	FR, SR	SR, BR,	BR, HB	MF, B3

N*ote*: *FR = Front row*, *SR = Second row*, *BR = Back row*, *HB = Half backs*, *MF = Midfield*, *B3 = Back three*.

The order of reporting within and across subsets relates to the means score for that dependent variable and is in the order of smallest first (i.e., the first position group reported in subset 1 is the smallest mean, and subset 1 has smaller means score than subset 2).

## Discussion

Our study provides the first insight into the movement demands of U20 international rugby matches played in 2014 and 2015. In addition, breakdown of the movements of positional groups in elite U20 internationals are also provided here for the first time. One of the uses for GPS during games is to compare and contrast positional roles to enable effective grouping of players for coaching and help to assess performance during matches [23]. Analysis revealed large differences between forwards and backs positions, further investigation highlighted variation within these groups and between individual player positions. Backs scored higher on all variables measured with the exception of number of accelerations 3-4m•s^-2^ (no difference between groups). These findings confirm that backs cover greater distances, however, it has been reported previously that forwards have a greater contact load [2, 5, 23].

Backs in our sample covered greater absolute distances (6.23 km) than forwards (5.37 km), these values are similar to those reported in European [4] and Pro 12 rugby [24], higher than those reported in Super Rugby in the 2008–09 season [25], but lower than values reported for the English Premiership [1], and a more recent super rugby study [6]. In relative terms, U20 backs also covered more distance per minute (69.1m•min^-1^) than forwards (61.5 m•min^-1^), again these values are slightly higher than those from European cup games (60.4 m•min^-1^ & 67.8 m•min^-1^) [4] and slightly less than the values reported in Premiership rugby (64.6 m•min^-1^ & 71.1 m•min^-1^ for forwards and back respectively) [1]. Interestingly, in the work of Reardon and colleagues [24], players covered similar absolute distances (6.23 v 6.17km for backs, 5.37 v 5.64 for forwards) to the players in the current study, however, they reported relative distances of 71.6m•min^-1^ for forwards and 81.0 m•min^-1^ for backs which is far greater than any research published on professional northern hemisphere rugby [1, 2, 4, 5], but similar to a recent study from a southern hemisphere club [6]. This difference in relative distance (but similar absolute distance) could be a result of a different methodology in how “game time” is defined, or a reflection of the style of play in the team they monitored. Other methodological issues may help to explain differences with previous GPS research. For example, some researchers only measured 1 back and 1 forward during a single match [5, 26]. This however, does not explain differences in our study compared to Cahill et al., [1] who conducted a comprehensive analysis of English Premiership rugby with 276 GPS data files. The vast majority of U20 players will come from Premiership clubs and the differences shown might reflect their status within that squad, i.e. junior/fringe first team players, and may not be deemed ready for the rigours of this level of competition. Furthermore, the sampling frequency (e.g. 1 and 5 Hz units) may account for differences between the current study and previous investigations. Other potential explanations for differences in movement characteristics, which need to be fully investigated are; differing playing standards, ball in play time, the team assessed, opponents’ tactics, refereeing, weather conditions and the period of the season from which games were assessed.

Our findings support numerous previous studies that demonstrate varying movement demands of different positional groups [1, 2, 6, 23]. For example, this study found that for measures of total distance, HSR, HSR•min^-1^, HML distance, HML Efforts, Sprints & Sprints•min^-1^, FR players were not significantly different to SR but significantly different to all other playing groups. Cahill and co-workers [1] reported similar findings for these positional groups, possibly due to the primary roles of these positions being to contest possession at set-pieces and break-downs [27, 28], therefore engaging in more high intensity static activity than that of the Backs [3]. For example, front five forwards are subjected to greater forces during scrummaging [29] which may result in transient fatigue; whereby there is a reduction in high-intensity activity performed immediately following an intense bout, with a subsequent recovery later in performance [30]. This may be characterised by reduced locomotive patterns compared to loose forwards following a scrum. Thus, despite advances in inertial sensor technology, which may help to characterise the movement patterns of rugby players, further research is required to quantify the physiological demand of performance. Monitoring player movements directly after intense static (e.g. Scrum or Maul) activity compared to movements not preceded by such an activity may give a useful indication of the level of transient fatigue.

The number of sprints performed by backs was over double that of the forwards (26.44 ± 7.47 vs 11.15 ± 5.06), with the MF and B3 performing the most (27.86 ± 6.32 & 28.89 ± 6.11 respectively) and the FR and SR performing the least (8.73 ± 4.52 & 10.0 ± 3.27 respectively). The B3 and MF positions covered significantly more HSR distance than the HB group and all forward groups. HBs covered more HSR than SR and FR but not BR (see Table 1). These trends are very similar to those reported by Jones et al., [2] but in opposition to the work of Cahill et al., [1] who reported forwards covered greater sprinting distances than backs. Other studies in rugby union that have used the same HSR thresholds as the current study have reported lower values for forwards (284.19 ± 134.85m vs 231 ± 167m) and backs (656.93 ± 182.70m vs 509 ± 150m) [4]. Jones and colleagues [2] also used the same positional groups when investigating match demands. The professional players in their research for each positional group covered less HSR distance than the U20 international players in the current study. This may reflect the different game demands, the progression of the game, or could be due to the different brand of GPS sensor used. Regardless, the high HSR distance achieved by the backs will likely lead to high levels of muscle damage, and recovery strategies should be employed to lessen the considerable disturbance in neuromuscular function seen up to 60 hrs post game [31]. Researchers have been exploring the use of relative speed zones as a fairer way to compare players [1, 24, 32, 33]. A number of different methods and thresholds have been used which is problematic for comparative purposes. Researchers have used a % of maximum running velocity, however some studies have utilised 50% of Vmax for the lower threshold of HSR [1, 32] while others have used 60% [24]. In a study using soccer players, the speed at the 2^nd^ ventilatory threshold was used for the assessment of HSR distance, which equated to over 80% maximum treadmill running speed. A substantial underestimation when using absolute compared to relative zones for determining HSR distance was found in this cohort. The amount of HSR performed when using relative compared to absolute zones has been reported to be underestimated in slower players and overestimated in faster ones according to the work of Gabbett [32]. Reardon and co-workers [24] reported that the use of absolute rather than relative speed zones led to some positions being under estimated (prop, second row) some over-estimated (flanker, No. 8, outside half, wing & fullback), and for others there was no difference between the methods for HSR distance (hooker, scum half, centre). Cahill et al., [1] reported in contrary to most of the published literature that forwards performed more sprinting than backs. However, the Vmax recorded in their study was determined during match play. The nature of the game could indeed prohibit players eliciting true maximal speed therefore sprinting distances could be over-estimated in some positions (particularly the Forwards). This should be considered when interpreting these findings. Given the sometimes conflicting and variable nature of different methods, reporting both absolute and relative derived values may be most useful [25], however this is problematic in an international camp where limited time is available for testing athletes.

Although some studies group hookers with the back row [9, 27, 34], in a recent cluster analysis of international players, Quarrie and co-workers [23] established that the hooker is better fitted to being grouped with props or second rows. They also suggested the scrum half should be a separate position and not grouped because of their more unique role. However due to a low number of scrum halves and outside halves making the inclusion criteria, in our study they were grouped as half backs to provide meaningful comparison to other positional groups. The FR and HB position had the lowest playing times and provided the least amount of files as they were the most frequently substituted group (before 60 mins), the only significant difference in playing time however, was between FR and MF group who had the greatest playing time and were the least substituted group.

In conclusion, this study is the first to describe the physical demands of the various positional groups during U20 international matches. Forwards are involved in a substantially higher number of collisions than backs, however, backs cover greater HSR distances, which should be considered when programing training and planning recovery activities post game. The distances covered during these matches is similar to research published on northern hemisphere club rugby but with this international team covering more HSR distance, suggesting that U20 internationals may be adequate in preparing players for the locomotor demands of professional club rugby.
